# The Role of N6-Methyladenosine in Mitochondrial Dysfunction and Pathology

**DOI:** 10.3390/ijms26083624

**Published:** 2025-04-11

**Authors:** Wenxin Yan, Saqirile, Ke Li, Kexin Li, Changshan Wang

**Affiliations:** School of Life Science, Inner Mongolia University, Hohhot 010020, China; 15065525077@163.com (W.Y.); sqrl1009@163.com (S.); like990614@163.com (K.L.); kexinli@mail.imu.edu.cn (K.L.)

**Keywords:** mitochondrial dysfunction, N6-methydenosine, cancer

## Abstract

Mitochondria are indispensable in cells and play crucial roles in maintaining cellular homeostasis, energy production, and regulating cell death. Mitochondrial dysfunction has various manifestations, causing different diseases by affecting the diverse functions of mitochondria in the body. Previous studies have mainly focused on mitochondrial-related diseases caused by nuclear gene mutations or mitochondrial gene mutations, or mitochondrial dysfunction resulting from epigenetic regulation, such as DNA and histone modification. In recent years, as a popular research area, m^6^A has been involved in a variety of important processes under physiological and pathological conditions. However, there are few summaries on how RNA methylation, especially m^6^A RNA methylation, affects mitochondrial function. Additionally, the role of m^6^A in pathology through influencing mitochondrial function may provide us with a new perspective on disease treatment. In this review, we summarize several manifestations of mitochondrial dysfunction and compile examples from recent years of how m^6^A affects mitochondrial function and its role in some diseases.

## 1. Introduction

Mitochondria, as dynamic organelles with a double-membrane structure, perform various functions in cells, such as maintaining various cellular homeostases, generating energy through oxidative phosphorylation, and regulating cell death [1]. Mitochondrial dysfunction directly disrupts core cellular processes, such as energy metabolism, redox balance, and apoptosis regulation, serving as a central driver of diverse pathologies. For example, mitochondrial dysfunction is considered one of the major causes of cardiovascular diseases, with factors such as an impaired mitophagy, reduced oxidative phosphorylation capacity, and increased production of reactive oxygen species (ROS) all contributing to their development [2]. Mitochondrial dysfunction, such as damage to the electron transport chain, the increased production of ROS, and alterations in mitochondrial dynamics, is a prominent feature of neurodegenerative diseases [3]. In addition, changes in mitochondrial fusion and fission can also lead to the occurrence of tumors and affect tumor metabolism and proliferation [4]. In summary, it can be found that the phenomenon of mitochondrial dysfunction occurs in a variety of diseases.

As an important part of epigenetics, RNA methylation has so far discovered over 150 types of RNA chemical modifications. Among them, methylation at the N6 position of adenine, namely N6-methyladenosine (m^6^A), which was first discovered in mRNA in 1974, is the most abundant RNA modification in eukaryotes [5]. Studies have found that m^6^A is enriched around stop codons, in the 3′ untranslated regions (3′UTRs), and near long internal exons, accounting for approximately 0.1% to 0.4% of the total adenosine residues in cellular RNA [6]. M^6^A is involved in regulating nearly every aspect of RNA biology, including RNA splicing, export, stability, and translation [7].

**Figure 1:**
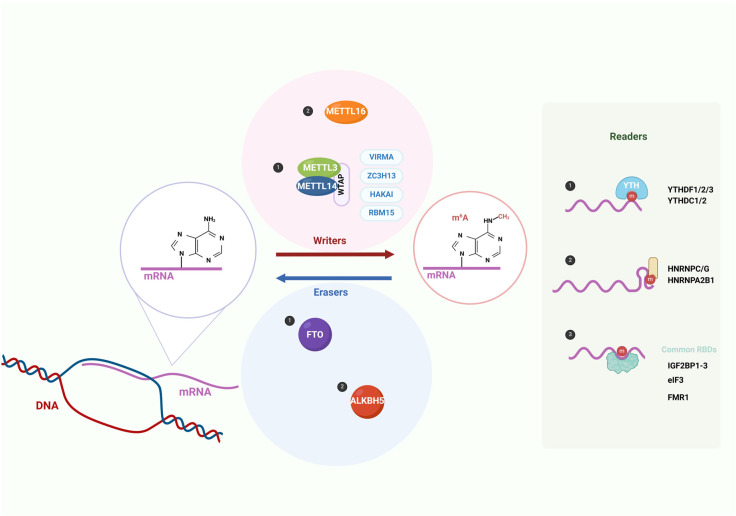
M^6^A regulators (created with BioRender.com). M^6^A methylation is a dynamic process, and there are three types of regulators involved in this process: writers, erasers, and readers. Writers mainly include the methyltransferase complex with METTL3, METTL14, and WTAP as the core, as well as METTL16. FTO and ALKBH5 are two demethylases, which are erasers. Readers play an important role in determining the fate of target RNAs, mainly including YT521-B homology (YTH) domain-containing protein families, IGF2 mRNA-binding protein (IGF2BP) families, heterogeneous nuclear ribonucleoprotein (HNRNP) protein families, and eukaryotic initiation factor (eIF) 3.

As shown in Figure 1, m^6^A methylation is a dynamic process that can be assembled by the methyltransferase complex (MTC) and removed by demethylases [8]. The regulators involved in this dynamic process can be classified into three categories: methyltransferase complex (writers), demethylases (erasers), and RNA-binding proteins (readers) [9].

METTL3, METTL14, and WTAP, as core components of the methyltransferase complex, play a major role in mediating m^6^A deposition on RNA through the stable heterodimer formed by METTL3 and METTL14. WTAP affects m^6^A deposition by interacting with the METTL3 METTL14 complex, but it does not have methylation activity itself [10]. Additionally, METTL16, as a new writer, targets pre-mRNAs, lncRNAs, and ncRNAs and methylates A43 of U6-snRNA [11].

FTO and ALKBH5, as two demethylases, can oxidatively remove m^6^A modifications on RNA (Table 1). FTO, as a member of the non-heme Fe(II) and α-KG-dependent dioxygenase AlkB family proteins, is located in the nucleus and cytoplasm of different mammalian cells. It has different structural features from other AlkB family proteins, including an additional C-terminal domain. The structural features of FTO enable it to demethylate different RNA substrates by binding to different partner proteins in different cellular environments [12,13]. Unlike FTO, ALKBH5 is only responsible for the removal of m^6^A on ssRNAs and is mainly located in the nucleus. Due to its localization in nuclear speckles that facilitate the assembly of mRNA-processing factors, it is speculated that nascent nuclear RNA is the main substrate of ALKBH5 [14,15].

Readers can recognize m^6^A and bind to it, playing a crucial role in determining the fate of target RNAs. These readers include YT521-B homology (YTH) domain-containing protein families, IGF2 mRNA-binding protein (IGF2BP) families, heterogeneous nuclear ribonucleoprotein (HNRNP) protein families, and eukaryotic initiation factor (eIF) 3 [16]. YTHDF2 plays a major role in RNA degradation, which induces transcript degradation by selectively recognizing and binding to m^6^A-modified mRNA and locating the bound mRNA to mRNA decay sites [17]. YTHDF1 enhances the translation efficiency of m^6^A-modified mRNA by interacting with initiation factors [18]. YTHDF3 cooperates with YTHDF1 to promote RNA translation and affects the YTHDF2-mediated degradation of methylated mRNA. As a partner of YTHDF1 and YTHDF2, YTHDF3 significantly influences their functions [19]. YTHDC1, as a nuclear reader, participates in alternative splicing [20]. Compared to other family members, YTHDC2 is the only one that contains helicase domains and multiple RNA-binding domains, and it has multiple functions, such as potentially increasing the translation efficiency of its target RNA and playing a crucial role in fertility [21,22]. The YTH-domain-containing proteins are widely involved in post-transcriptional regulation, participating in the regulation of target mRNA splicing, promoting translation, or promoting RNA degradation [23].

IGF2BP promotes the stability of target mRNA by inhibiting the degradation of m^6^A-containing mRNA and promoting its translation, as well as enhancing mRNA storage [24]. HNRNPC, as a nuclear RNA-binding protein, binds to nascent RNA transcripts by recognizing m^6^A sites, thereby affecting the stability, alternative splicing, and translation of target pre-mRNA [25]. HNRNPA2B1, together with METTL3, regulates the alternative splicing of target RNAs and the processing of nuclear pri-miRNA [26]. Eukaryotic initiation factor 3 (eIF3) promotes m^6^A-mediated cap-independent translation [27].

M^6^A RNA methylation is widely involved in the regulation of physiological and pathological processes, including cancer. Multiple studies have shown that m^6^A is involved in the regulation of tumors, but there is still no definite conclusion on how m^6^A affects mitochondrial function.

**Table 1:** The function of m^6^A regulators in RNA metabolism.

Category	Factor	Full Name	Function	Reference
Writers	METTL3	Methyltransferase-like 3	As the catalytic core of the m^6^A methyltransferase complex, it catalyzes m^6^A modification.	[28]
	METTL14	Methyltransferase-like 14	Acts as an RNA binding platform within the m^6^A methyltransferase complex and forms a heterodimer with METTL3 to catalyze m^6^A modification.	[5,28]
	WTAP	Wilms tumor 1- associated protein	As a regulatory subunit in the m^6^A methyltransferase complex, it interacts with METTL3 and METTL14 and directs them to nuclear speckles.	[29,30]
	METTL16	Methyltransferase-like 16	A single-component methyltransferase that can methylate U6-snRNA, the MALAT1 long non-coding RNA, and the MAT2A pre-mRNA.	[31,32]
Writers	VIRMA (KIAA1429)	Vir-like m^6^A methyltransferase-associated	Recruits the core components of the methyltransferase METTL3/METTL14/WTAP to specific regions for selective methylation.	[33]
	RBM15/ RBM15B	RNA binding motif protein 15/ RNA binding motif protein 15B	Binds to the m^6^A methyltransferase complex and guides it to specific RNA sites.	[34]
	ZC3H13	Zinc finger CCCH-type containing 13	Controls the nuclear localization of the Zc3h13–WTAP–Virilizer–Hakai complex and promotes m^6^A methylation.	[35]
	HAKAI (CBLL1)	Cbl Proto-Oncogene Like 1	Maintains the stability of the m^6^A methyltransferase complex.	[36]
Erasers	FTO	Fat mass and obesity-associated	Removes m^6^A modification and promotes mRNA splicing.	[37]
	ALKBH5	AlkB homolog 5	Removes m^6^A modification on nuclear RNA, thereby regulating nuclear mRNA export.	[14]
Readers	YTHDF1	YTH N6-methyladenosine RNA-binding protein 1	Promotes the translation initiation of m^6^A-modified mRNA.	[18]
	YTHDF2	YTH N6-methyladenosine RNA-binding protein 2	Promotes the degradation of m^6^A-modified mRNA.	[17]
	YTHDF3	YTH N6-methyladenosine RNA-binding protein 3	As a partner of YTHDF1 and YTHDF2, it interacts with YTHDF1/YTHDF2 to promote mRNA translation or degradation.	[19]
	YTHDC1	YTH domain containing 1	Promotes mRNA splicing.	[20]
	YTHDC2	YTH domain containing 2	Increases the translation efficiency of the target mRNA.	[21,22]
	IGF2BP1/2/3	Insulin-like growth factor 2 mRNA-binding protein 1/2/3	Promotes the stability of the target mRNA and facilitates its translation.	[24]
	HNRNPC	Heterogeneous nuclear ribonucleoprotein C	Affects the stability, alternative splicing, and translation of pre-mRNA.	[25]
	HNRNPA2B1	Heterogeneous nuclear ribonucleoprotein A2/B1	Promotes the processing of nuclear pri-miRNA and mRNA splicing.	[26]
	eIF3	Eukaryotic initiation factor 3	Promotes m^6^A-mediated cap-independent translation.	[27]

## 2. Mitochondrial Function and Dysregulation

### 2.1. Mitochondrial Structure and Function

**Figure 2:**
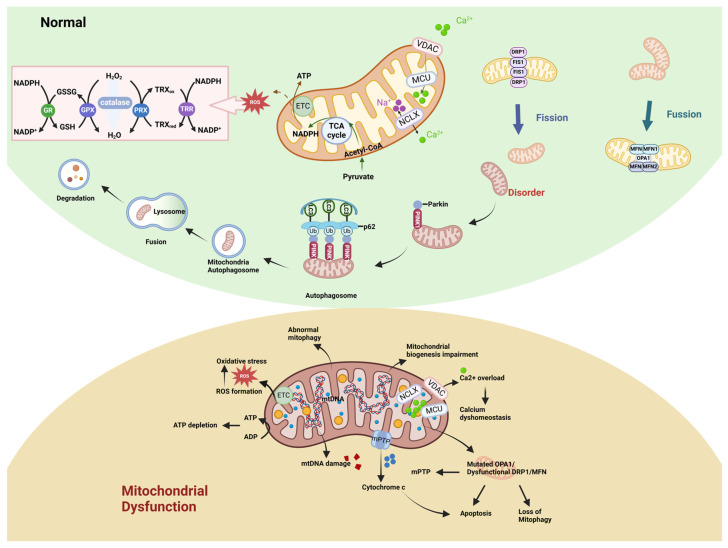
Comparison between normal mitochondrial function and mitochondrial dysfunction (created with BioRender.com). Mitochondria are multifunctional organelles that play crucial roles in maintaining cellular ROS and Ca^2+^ homeostasis, ATP production, and regulating cell death; when mitochondrial dysfunction occurs, there will be decrease in ATP production, damage to mitochondrial DNA, oxidative stress, imbalance of calcium homeostasis, abnormal mitochondrial dynamics, and occurrence of cell death.

The α-proteobacterium engulfed by the precursor of modern eukaryotic cells is the ancestor of mitochondria [38]. During the evolution of bacteria into mitochondria, a portion of the genomic material was retained, known as mitochondrial DNA (mtDNA), which replicates in a semi-conservative manner and encodes some essential RNAs and proteins [39]. Mitochondria are organelles with a double-membrane structure consisting of four domains: the outer membrane, the inner membrane, the intermembrane space, and the mitochondrial matrix. The inner membrane folds inward to form cristae, which are the main site for oxidative phosphorylation [40]. Large biomolecules are degraded through the tricarboxylic acid (TCA) cycle and β-oxidation to produce NADH and FADH2. NADH and FADH2 dehydrogenate to form high-energy electrons and protons. The high-energy electrons are transferred along the electron transport chain (ETC) to O_2_ and gradually release energy, while protons are directionally transferred across the inner mitochondrial membrane (IMM) from the matrix side to the intermembrane space, finally forming a transmembrane potential difference and pH difference, known as the proton motive force, which then drives the rotational catalysis of ATP synthase to generate ATP. This process is called oxidative phosphorylation (OXPHOS) [41,42,43,44]. Mitochondria are multifunctional organelles. Besides OXPHOS, they also participate in amino acid metabolism, lipid synthesis, lipid oxidation, ROS generation, redox homeostasis, signal transduction, and apoptosis. These functions are crucial for maintaining multicellular life [45]. Mitochondrial dysfunction leads to reduced ATP synthesis, oxidative stress, inflammatory responses, and increased ROS levels, and may trigger various diseases, including neurodegenerative diseases, cardiomyopathy, metabolic syndrome, cancer, and obesity [46,47,48,49]. The causes of mitochondrial dysfunction include impaired mitochondrial biogenesis (Figure 2), increased ROS production, defective mitophagy, transport impairment, ETC dysfunction, changes in mitochondrial dynamics, and calcium imbalance or a combination of these factors [50].

### 2.2. Manifestations of Mitochondrial Dysfunction

#### 2.2.1. Abnormal Mitochondrial Energy Metabolism and Impaired Biogenesis

Mitochondria can generate ATP through OXPHOS, as described in the process of OXPHOS outlined above. Mitochondrial diseases are caused by mitochondrial dysfunction resulting from nuclear gene mutations or mitochondrial gene mutations, and they are some of the most common genetic diseases. Their main feature is mitochondrial OXPHOS deficiency [51]. Only a small number of the proteins in mitochondria are encoded by mtDNA, which are the core components of OXPHOS complexes in the IMM, including seven polypeptides (ND1, ND2, ND3, ND4, ND4L, ND5, and ND6) in complex I, one polypeptide (cytochrome b) in complex III, three polypeptides (COI, COII, and COIII) in complex IV, and two polypeptides (ATP6 and ATP8) in complex V, totaling thirteen mitochondrial proteins. In addition, mtDNA also encodes 22 mt-tRNAs and 2 mt-rRNAs [52,53]. Approximately 99% of mitochondrial proteins are encoded by nuclear genes [53]. Moreover, the absence of or defects in enzymes catalyzing different steps in the ETC can also lead to mitochondrial dysfunction.

The electrochemical gradient (membrane potential) of the IMM that accompanies OXPHOS is a fundamental property of mitochondria and a major driving force for protein transport [54]. The steady state of the mitochondrial membrane potential is an important prerequisite for mitochondrial health and the maintenance of normal cellular and tissue functions [55].

Mitochondrial biogenesis (MB) is a complex and multifaceted regeneration process that controls mitochondrial self-renewal, replacing damaged mitochondria with healthy ones, and maintaining mtDNA to promote cellular homeostasis. The main events include the mitochondrial fusion, reduction in ROS, restoration of mitochondrial membrane potential, and increased expression of OXPHOS proteins. Impaired MB can lead to mitochondrial and cellular dysfunction [56,57]. Many studies have shown that peroxisome proliferator-activated receptor gamma coactivator 1 alpha and 1 beta (PGC-1α and PGC-1β) and PGC-1-related coactivator (PRC) play significant roles in mitochondrial biogenesis (Table 2). As downstream factors of these coactivators, the nuclear transcription factors NRF1 and NRF2 regulate the expression of mitochondrial respiratory complex subunits, and mitochondrial transcription factor A (TFAM) is essential for mtDNA transcription and replication [58]. PGC-1α coordinates the expression of essential biogenesis proteins through NRF1 and NRF2 and drives the production of mitochondrial proteins, such as complex V and cytochrome c (Cyt c). PGC-1α is currently considered the main regulator of MB [59]. Various signaling cascades regulate MB by influencing the PGC-1α-NRF1/2-TFAM pathway. For example, an increase in AMP will activate AMPK, and AMPK increases the expression of PGC-1α and TFAM by phosphorylating PGC-1α. After AMP is converted into cAMP, cAMP can regulate PGC-1α through the cAMP-PKA-CREB pathway. Ca^2+^ increases the activity and expression of PGC-1α by stimulating calcium/calmodulin-dependent protein kinase (CaMK) to phosphorylate p38 mitogen-activated protein kinase (p38 MAPK). In addition, CaMK can also stimulate PGC-1α through CREB. An increase in the level of NAD+ leads to the deacetylation of *PGC-1α* by Sirtuin 1 (Sirt1), activating PGC-1α [60].

#### 2.2.2. Alterations in Mitochondrial Dynamics

Mitochondrial dynamics encompass regulated processes (fission, fusion, mitophagy, and trafficking) that maintain mitochondrial morphology, quantity, and functional integrity. These adaptive mechanisms underpin mitochondria’s roles in metabolic and signaling networks. Dysregulation disrupts mitochondrial homeostasis, resulting in disease pathogenesis [61,62].

Fission is essential for providing a certain number of mitochondria during cell growth and division and can conduct the quality control of mitochondria by eliminating damaged or dysfunctional mitochondria and promoting apoptosis in severely stressed cells [63]. In mammals, the process of mitochondrial fission is mediated by the GTPase dynamin-related protein DRP1 (or DLP1), which is mainly located in the cytoplasm and recruited to mitochondria by MID49, MID51, and MFF, forming a helix around the mitochondria, and the helix contracts to sever the inner and outer membranes [64,65]. Mitochondrial fission is regulated by post-translational modifications of DRP1. DRP1 exists in an inactive state in the cytoplasm, and its activation is regulated by phosphorylation, SUMOylation, ubiquitination, and S-nitrosylation modification [66,67]. Defects in mitochondrial fission may lead to mitochondrial dysfunction, further causing related diseases with the accumulation of damaged mitochondria. This accumulation of damaged mitochondria has a negative impact on the components of the ETC and inhibits ATP production, ultimately leading to cell death [68,69].

During the fusion process, the contents of mitochondria are mixed and exchanged; this process can redistribute mtDNA, mRNA, and proteins to compensate for defective mitochondria, promote the ability of OXPHOS, and maintain mitochondrial function [63,70]. Mitochondrial fusion is a two-step process involving three dynamin-related GTPases. The fusion of the outer mitochondrial membrane (OMM) is mediated by mitofusin 1 (MFN1) and mitofusin 2 (MFN2), while the fusion of the IMM is mediated by optic atrophy protein 1 (OPA1) [71,72]. OPA1, together with MFN1, mediates mitochondrial fusion. In addition, OPA1 also plays an important role in regulating the shape of cristae, the arrangement of ETC supercomplexes, and the control of Cyt c release [73]. The absence of OPA1 leads to the collapse of the mitochondrial network and damage to mitochondrial morphology, and it eventually promotes apoptosis [74]. The deficiency of MFN1 has a relatively minor impact on mitochondrial function, while the deficiency of MFN2 leads to an increase in proton leakage, a decrease in the mitochondrial membrane potential (ΔΨ_m_), and the generation of ROS [75]. During the fusion process, the mixing of the matrix and inner membrane enables the entire mitochondrial compartment to participate in respiration, maximizing ATP synthesis. When there are fusion defects in mitochondria, it leads to mitochondrial dysfunction and the loss of respiratory function [76].

#### 2.2.3. Abnormal Mitophagy

Cells remove dysfunctional or redundant mitochondria through mitophagy, which is essential for maintaining a healthy mitochondrial network [77]. In mammals, mitophagy has a complex and precise mechanism and regulatory mode, which can generally be divided into two categories: ubiquitin (Ub)-dependent pathways and Ub-independent pathways.

Ub-dependent pathways promote mitophagy through the ubiquitination of mitochondrial surface proteins. The phosphatase and tensin homolog (PTEN)-induced putative kinase 1 (PINK1)–Parkin pathway regulates mitophagy in mammals. In this process, the depolarization of the IMM of damaged mitochondria prevents PINK1 from being degraded and allows it to accumulate in the OMM. PINK1 recruits the E3 ubiquitin ligase Parkin and phosphorylates ubiquitin at the Ser65 site, activating Parkin ubiquitin ligase activity. The ubiquitination of multiple OMM proteins (such as MFN1, MFN2, and VDAC1) mediated by Parkin enables them to be recognized by autophagy adaptor proteins or degraded by the proteasome. At the same time, it attracts autophagy receptors (such as p62 and optineurin), and LC3 autophagy-related proteins bind to damaged mitochondria through these receptors, promoting the formation of autophagosomes. Autophagosomes fuse with lysosomes to form autolysosomes, thereby eliminating damaged mitochondria [77,78].

Ub-independent pathways refer to receptor proteins on the OMM containing the LC3-interacting region (LIR), which can directly bind to LC3 to initiate mitophagy without ubiquitination. These receptors include Nip3-like protein X (NIX), also known as BCL2-interacting protein 3 like (BNIP3L) receptor, BCL2-interacting protein 3 (BNIP3) receptor, FUN14 domain containing 1 (FUNDC1) receptor, etc. [79]. Both NIX and BNIP3 can directly bind to LC3 through the BH3 domain to induce mitophagy, and phosphorylation enhances their binding to LC3; FUNDC1 induces Parkin-independent mitophagy by interacting with LC3 under hypoxic conditions. Under normal physiological conditions, the Tyr18 and Ser13 sites of FUNDC1 are phosphorylated by Src kinase and CK2 kinase, respectively, preventing mitophagy. During the process of mitophagy, Ser13 is dephosphorylated, and Ser17 is phosphorylated by ULK1 to activate FUNDC1. In addition, mitophagy receptors can also promote the fission of damaged organelles by recruiting mitochondrial-dynamics-related proteins, such as DRP1 and OPA1, on the mitochondrial surface [80,81].

Mitophagy is meticulously coordinated by adaptive molecular circuits, which calibrate organelle turnover to metabolic flux. The breakdown of these circuits, through aberrant phosphorylation or defective cargo recognition, unleashes irreversible mitochondrial damage, promoting aging-associated and oncogenic trajectories. Various diseases, such as tumorigenesis, cardiovascular diseases, neurodegenerative diseases, and chronic inflammation, are associated with mitochondrial dysfunction and abnormal mitochondrial content [82,83]. When mitochondrial autophagy is insufficient, it leads to mitochondrial dysfunction and the accumulation of a large number of damaged mitochondria within the cell, further damaging the cell; conversely, highly activated mitochondrial autophagy can cause the excessive degradation of normal mitochondria, thereby affecting the cell’s energy supply and leading to cell death [60].

#### 2.2.4. Imbalance Between Generation and Clearance of Mitochondrial ROS

ROS are generated when a single electron is transferred from a redox donor to molecular oxygen (O_2_), and this process produces the anionic free radical superoxide, which can also be converted into hydrogen peroxide by superoxide dismutase (SOD). Electrons are transferred sequentially through the ETC to complex IV, where O_2_ acts as the final electron acceptor. This process drives the reduction of O_2_ to H_2_O via coupling with H^+^ in the mitochondrial matrix. However, electrons can also prematurely react with oxygen at ETC sites to form superoxide or hydrogen peroxide [84]. So far, up to 16 sources of ROS have been found in mitochondria, among which 12 ROS-producing sites are related to nutrient oxidation, electron transfer, and OXPHOS. Complex I and complex III are the main sites of ROS production in mitochondria [85].

Low concentrations of mitochondrial H_2_O_2_ (one of the types of ROS) are involved in regulating various cellular functions, including cell differentiation, apoptosis, cell proliferation, T-cell activation, and stress signal transduction. However, high concentrations of H_2_O_2_ are destructive, so controlling its generation and overall concentration is crucial. The antioxidant system plays an important role in removing ROS [86]. H_2_O_2_ can be degraded by mitochondria through three systems: reduced glutathione (GSH), thioredoxin-2 (TRX2), and catalase (CAT) [87]. The GSH system degrades peroxides through glutathione peroxidase (GPX) and simultaneously converts GSH into glutathione disulfide (GSSG) [88]. The TRX2 system degrades hydrogen peroxide through peroxiredoxin (PRX), which reduces hydrogen peroxide to water while being oxidized and inactivated, and the inactivated PRX can be reactivated by mitochondrial TRX2 [89]. Studies have found that mitochondria in the liver and heart also contain catalase, which plays a major role in removing hydrogen peroxide when its concentration is higher than normal. The GSH system and PRX system may act as buffering systems to maintain a low level of hydrogen peroxide for mitochondrial redox signaling [90].

ROS are produced during normal cell metabolism. Low concentrations of ROS are necessary for normal physiological functions, participating in immune responses, and maintaining cellular homeostasis [91]. However, if the level of ROS in mitochondria is too high, it is harmful. ROS can damage DNA, proteins, and lipids, leading to mitochondrial dysfunction and various diseases [92]. For example, ROS can induce mtDNA mutations, causing mitochondrial dysfunction; attack mitochondrial membrane lipids, disrupting the integrity of the membrane; and oxidatively modify mitochondrial proteins, affecting their function and stability. Conversely, mitochondrial dysfunction can lead to the abnormal function of the ETC, increased electron leakage, excessive ROS production, and oxidative stress, further damaging mitochondria and other cellular components [93].

#### 2.2.5. Imbalance of Calcium Homeostasis

Ca^2+^ is an important signaling molecule in cells. Through the mitochondrial calcium uniporter (MCU) complex, cytoplasmic calcium in the cytoplasm enters the mitochondrial matrix and functions as a signal to regulate ATP production and metabolic fuel selection [94,95]. Ca^2+^ enters the mitochondrial intermembrane space (IMS) through the voltage-dependent anion channel (VDAC) located on the OMM and then enters the mitochondrial matrix through the MCU complex [96]. The MCU complex is located on the IMM and is composed of the mitochondrial calcium uniporter (MCU) and accessory proteins, such as mitochondrial calcium uptake 1/2 (MICU1/2), MCU regulator 1 (MCUR1), MCU dominant-negative β-subunit (MCUb), solute carrier 25A23 (SLC25A23), and essential MCU regulator (EMRE). The mitochondrial membrane potential, cytosolic Ca^2+^ gradient, oxidants, pH, and ions are involved in regulating the activity of the MCU complex [97]. The extrusion of Ca^2+^ from mitochondria is mainly mediated by the mitochondrial Na^+^/Ca^2+^/Li^+^ exchanger (NCLX). One cycle involves the import of three Na^+^ and the extrusion of one Ca^2+^ from the mitochondria. PTEN-induced putative kinase 1 (PINK1) acts as a regulator of mitochondrial Ca^2+^ and regulates the NCLX by mediating the phosphorylation of the NCLX through protein kinase A (PKA) [98]. Mitochondrial Ca^2+^ homeostasis plays a crucial role in mitochondrial energy metabolism, apoptosis, and ROS production [99]. A low level of matrix-free calcium can improve OXPHOS and activate matrix dehydrogenases, while calcium overload inhibits OXPHOS and reduces ATP synthesis through multiple mechanisms, and high concentrations of calcium phosphate precipitates can disrupt the stability of the mitochondrial cristae network, leading to mitochondrial rupture and fragmentation [100]. In addition, calcium can affect the activities of metabolic enzymes in the TCA cycle, such as pyruvate dehydrogenase, isocitrate dehydrogenase, and α-ketoglutarate dehydrogenase [101]. If an excessive amount of Ca^2+^ flows into mitochondria, it will lead to mitochondrial outer membrane permeabilization, and pro-apoptotic factors, such as Cyt c and apoptosis-inducing factor, will be released into the cytoplasm, causing cell death [102].

#### 2.2.6. Cell Death

Mitochondria execute important functions in the process of intrinsic apoptosis; during this process, the B-cell lymphoma gene 2 (BCL-2) protein family exerts significant regulatory effects. The oligomerization pro-apoptotic factors Bak and Bax in the BCL-2 family interact with VDAC on the OMM, regulate the permeability of the OMM, and release Cyt c into the cytoplasm, and subsequently trigger the caspase cascade, leading to apoptosis [103,104]. Under normal circumstances, BAX and BAK are in an inactive state, with BAX located in the cytoplasm and BAK in the mitochondria. Both can freely shuttle between the mitochondria and cytoplasm. During apoptosis, BAX and BAK are directly activated by binding to the BH3 protein subfamily, causing them to anchor to the OMM and dimerize. These dimers then further oligomerize, creating lipid pores on the OMM and changing the permeability of the OMM [105]. The mitochondrial permeability transition pore (mPTP) is a conductive channel located on the IMM and plays a significant role in cell death. The mPTP will be activated when there is calcium overload and an increase in ROS, disrupting the mitochondrial membrane potential and leading to cell death [106].

The progressive mitochondrial dysfunction that occurs during aging may predispose cells to necroptosis. For instance, mitochondrial ROS can increase the autophosphorylation of receptor-interacting protein kinase 1 (RIPK1), recruit RIPK3, and promote the assembly of the necrosome complex, thus facilitating the occurrence of necroptosis [107]. Recent studies have shown that RIPK3 promotes aerobic respiration and mitochondrial ROS production by phosphorylating and activating the E3 subunit of the pyruvate dehydrogenase complex, indicating an association between mitochondrial instability and necroptosis [108].

Ferroptosis is closely associated with mitochondrial dysfunction. The excessive activation of the ETC leads to an increased release of mitochondrial ROS, enhancing the oxidation of polyunsaturated fatty acids (PUFAs) and increasing PUFA synthesis by inhibiting the AMPK pathway, resulting in increased lipid peroxidation. Additionally, damaged mitochondria can also indirectly downregulate glutathione-dependent peroxidase (GPX4) through the inhibition of the integrated stress response mediated by EIF2α phosphorylation. The breakdown of glutamine and the TCA cycle in mitochondria can further promote ferroptosis by driving the activity of the ETC. As mentioned above, mitochondria can promote ferroptosis by playing various roles in bioenergetics, biosynthesis, and ROS regulation [109,110,111]. In the cells undergoing ferroptosis, there are huge morphological changes in mitochondria, such as contracted mitochondria with increased membrane density, enlarged cristae, and a ruptured OMM [112]. The role of mitochondria in ferroptosis is conditional: it plays a key role in ferroptosis induced by cysteine deprivation. If ferroptosis is induced by the inhibition of GPX4 activity, cells will adopt a form of ferroptosis independent of mitochondrial function [113].

ROS produced in mitochondria activate the NLRP3 inflammasome, and caspase-1 is activated to form mature caspase-1, which leads to the maturation of interleukin (IL)-18 and IL-1β and the cleavage of GSDMD to generate N-GSDMD, inducing pyroptosis. Mitophagy can reduce the activation of the NLRP3 inflammasome by eliminating damaged mitochondria and reducing ROS production. However, the activation of caspase-1 inhibits mitophagy and increases mitochondrial damage [114,115]. Moreover, mitochondrial damage caused by Ca^2+^ overload generates a large amount of ROS, which can also lead to the activation of the NLRP3 inflammasome and pyroptosis [107].

#### 2.2.7. Mitochondria-Targeted Therapy in Diseases

Mitochondria-based disease treatments have become an emerging trend. A wide range of studies have developed mitochondria-targeted delivery systems for disease therapy. For instance, mitochondriotropic particulate carriers, on the basis of liposomes, biodegradable polymers, and metals, target mitochondria through the incorporation of mitochondriotropic agents onto the surface and delivering therapeutic molecules to mitochondria to induce mitochondria dysfunction [116]. Derivatives of triphenylphosphonium (TPP) are lipophilic and have an affinity for mitochondria; by leveraging mitochondrial localization to enhance the therapeutic effect of diseases, they serve as effective cancer-targeting ligands [117]. Research based on amphipathic peptoids discovered two peptoids that showed highly efficient cell penetration and mitochondrial localization and might serve as potential transporters for the delivery of bioactive compounds, such as drugs, antioxidants, etc. [118]. Recently, a mitochondria-targeted RNAi nanoparticle (NP) platform, which targeted mitochondrial metabolism, was developed and employed in the treatment of breast cancer, and this system could successfully transport siRNA into the mitochondria to regulate mtDNA-encoded protein expression and inhibit tumor growth [119]. Researchers have also devised more novel methods to target mitochondria for ischemic stroke (IS) treatment; one study adopted nanomedicine-based brain targeting strategies and developed a magnetic-field-driven, mitochondria-targeted ceria (MMTCe) nanosystem. By using this system, the targeting of the damaged mitochondria was achieved, and the ischemic microenvironment was improved [120]. In addition, a platinum-based terminal-sensitive projectile (TSB) was developed to precisely target the mitochondria of tumor cells, significantly enhancing the sensitivity of tumor cells to platinum-based chemotherapeutic drugs [119]. Given the indispensable function of mitochondria in cell metabolism and growth, a growing body of evidence suggests that targeting mitochondria may ultimately be accepted as a promising strategy for cancer medication. Most studies focus on the nanoparticle-based delivery of mitochondria for cancer therapy [121,122,123,124,125].

**Table 2:** The role of factors in mitochondrial function.

Category	Factor	Full Name	Function	Reference
Mitochondrial Biogenesis	PGC-1α	Peroxisome proliferator-activated receptor gamma coactivator 1 alpha	Regulates processes such as mitochondrial biogenesis, fission, fusion, and mitophagy by modulating coactivators and downstream effector factors.	[126]
	PGC-1β	Peroxisome proliferator-activated receptor gamma coactivator 1 beta	Promotes mitochondrial biogenesis and is essential for normal OXPHOS and mitochondrial function.	[126]
	PRC	PGC-1-related coactivator	Activates the transcription factors NRF1 and NRF2, which are associated with the expression of the respiratory chain.	[127]
	TFAM	Mitochondrial transcription factor A	Essential for the transcription and replication of mtDNA.	[58]
Mitochondrial Dynamics	DRP1 (DLP1/ DNM1L)	The GTPase dynamin-related protein 1	Recruited from the cytoplasm to the outer mitochondrial membrane and mediate the process of mitochondrial fission.	[64,65]
	MID49	Mitochondrial dynamics proteins of 49	Recruits DRP1 and promotes mitochondrial fission.	[128]
	MID51	Mitochondrial dynamics proteins of 51	Recruits DRP1 and promotes mitochondrial fission.	[128]
	MFF	Mitochondrial fission factor	Acts as a DRP1 receptor on the mitochondrial membrane, recruits DRP1, and promotes mitochondrial fission.	[129]
	FIS1	Fission protein 1	Recruits DRP1 and facilitates mitochondrial fission.	[128]
	MTP18 (MTFP1)	Mitochondrial fission process 1	Maintains the mitochondrial morphology by regulating mitochondrial fission.	[130]
	MFN1	Mitofusin 1	Together with MFN2, mediates the fusion of the OMM in a GTP-dependent manner, coordinating the sequential fusion of the OMM and the IMM with the IMM fusion regulator OPA1.	[129]
	MFN2	Mitofusin 2	Together with MFN1, it mediates the fusion of the OMM in a GTP-dependent manner, mediates mitochondria–ER tethering, and transfers phosphatidylserine from ER to mitochondria.	[129]
	OPA1	Optic atrophy protein 1	Controls the fusion of the IMM and participates in processes such as regulating the shape of cristae, the arrangement of ETC supercomplexes, and the control of Cyt c release.	[73]
Mitophagy	PINK1	Phosphatase and tensin homolog (PTEN)-induced putative kinase 1	Recruits Parkin to initiate mitophagy and eliminate damaged mitochondria.	[130]
	Parkin	Parkin	E3 ubiquitin ligase, ubiquitinates multiple OMM proteins, recruits autophagy receptors to damaged mitochondria, and promotes mitophagy.	[130]
	BNIP3L (NIX)	BCL2-interacting protein 3 like	Participates in the process of mitophagy as a mitophagy receptor and promotes the formation of autophagosomes.	[131]
	BNIP3	BCL2-interacting protein 3	Induces mitophagy by binding to LC3 through the BH3 domain.	[132]
	FUNDC1	FUN14 domain containing 1	A ubiquitin-independent mitophagy receptor that can directly bind to LC3 to initiate mitophagy.	[133]
Mitochondrial Oxidative Stress	SOD	Superoxide dismutase	An antioxidant enzyme that converts superoxide anion radicals into hydrogen peroxide and oxygen.	[84]
	GPX	Glutathione peroxidase	An antioxidant enzyme family that utilizes reduced glutathione as an electron donor to catalyze the reduction of hydrogen peroxide or organic hydroperoxides to water or the corresponding alcohols.	[134]
	TRX2	Thioredoxin-2	Clears ROS in the cell through the TRX2/PRX system and regulates the apoptotic signaling pathway by inhibiting oxidative stress.	[135]
	PRX	Peroxiredoxin	A multifunctional enzyme that reduces peroxides through the cysteine residues at the active center and also acts as a redox signaling regulator, chaperone, and pro-inflammatory factor.	[136]
Calcium Homeostasis	MCU	Mitochondrial calcium uniporter	Forms a pore through which calcium ions enter the mitochondria and regulates the concentration of Ca^2+^ in the mitochondria.	[137,138]
	MICU1	Mitochondrial calcium uptake 1	A key regulatory factor for mitochondrial Ca^2+^ uptake. When the intracellular Ca^2+^ level is high, it promotes the influx of calcium ions into the mitochondria.	[139,140]
	MICU2	Mitochondrial calcium uptake 2	When the level of calcium ions outside the mitochondria is low, it turns off the activity of the MCU.	[138]
	MCUR1	MCU regulator 1	Acts as a scaffolding factor to bind the MCU and EMRE.	[141]
	MCUb	MCU dominant-negative β-subunit	An inhibitory subunit of the MCU complex that forms a multimer with the MCU to inhibit the influx of Ca^2+^.	[142]
	SCL25A23	Solute carrier 25A23	Participates in mitochondrial Ca^2+^ uptake and interacts with the MCU and MICU1 to enhance the activity of the MCU channel.	[143]
	EMRE	Essential MCU regulator	Activates the function of the MCU, increases the uptake of Ca^2+^, and maintains the MICU regulation of the MCU pore.	[137]
	NCLX	Mitochondrial Na^+^/Ca^2+^/Li^+^ exchanger	Mediates the efflux of mitochondrial Ca^2+^ using the entry of sodium ions into the mitochondria along their concentration gradient as the driving force; transports calcium ions out of the mitochondria to maintain mitochondrial calcium homeostasis.	[98]
Cell Death	BAK	BCL2-antagonist/killer	Together with BAX, mediates the permeabilization of the OMM in the mitochondrial pathway and promotes apoptosis.	[144]
	BAX	BCL2 Associated X Protein	Together with BAK, mediates the permeabilization of the OMM in the mitochondrial pathway and promotes apoptosis.	[144]
	RIPK1	Receptor-interacting protein kinase 1	A key mediator of the apoptotic, necroptotic, and inflammatory pathways that mediate necroptosis.	[145]
	RIPK3	Receptor-interacting protein kinase 3	Acts as a downstream mediator of RIPK1 to trigger necroptosis.	[146]
	GPX4	Glutathione-dependent peroxidase 4	A form of glutathione peroxidase that specifically catalyzes the conversion of lipid hydroperoxides into non-toxic lipid alcohols to alleviate ferroptosis.	[147]

## 3. M^6^A and Mitochondrial Dysfunction

### 3.1. Role of m^6^A in Influencing Mitochondrial Function in Disease

Nuclear-encoded genes, when regulated by epigenetics, can affect the expression of mitochondrial proteins, such as histone acetylation, as well as histone and DNA methylation [148]. However, the relationship between RNA methylation and mitochondrial function has not been systematically explained. This review will explore and summarize how m^6^A RNA methylation in epigenetics affects mitochondrial function.

Studies have found that m^6^A RNA methylation regulates mitochondrial function by promoting the translation of nuclear-encoded mitochondrial ETC subunit RNAs. After knocking out the RNA methyltransferase METTL14, the absence of m^6^A significantly downregulated metabolites related to energy metabolism and led to a significant decrease in the mitochondrial respiratory capacity and membrane potential. These functional defects are consistent with the reduced expression of mitochondrial ETC complexes, as well as the decreased assembly and activity of mitochondrial supercomplexes. Mechanistically, the absence of m^6^A reduces the translation efficiency by decreasing the binding of methylated RNAs encoding mitochondrial complex subunits to polysomes, but it has no effect on RNA stability. Moreover, it has been found that the regulation of mitochondrial complex RNA translation by m^6^A may be mediated by the YTHDF family of readers [149].

As an m^6^A demethylase, FTO regulates mitochondrial content by mediating the expression of genes related to mitochondrial fusion, fission, and biogenesis. Experimental results show that when FTO is overexpressed, genes involved in mitochondrial fusion (*MFN1/2* and *OPA1*) are upregulated, genes involved in mitochondrial fission (*FIS1*, *DRP1*, and *MTP18*) are downregulated, and the expression of genes involved in mitochondrial biogenesis (*PGC-1α* and *TFAM*) is inhibited. In summary, FTO may reduce mitochondrial content and ATP levels by promoting mitochondrial fusion and inhibiting mitochondrial fission [150,151,152]. As a positive regulator of mitophagy, FTO can promote mitophagy by regulating the level of the mitophagy receptor BNIP3 in an m^6^A-dependent manner. Mechanistically, the absence of FTO leads to an increase in the m^6^A modification level of *BNIP3*. YTHDF2 binds to *BNIP3* by recognizing m^6^A sites, promoting the degradation of its mRNA and a reduction in BNIP3 protein levels. FTO promotes mitophagy by increasing the stability of *BNIP3* mRNA [153]. After cerebral ischemia/reperfusion (I/R), a low expression of FTO was found in the brain tissue, the level of m^6^A modification on *NRF2* mRNA increased, and the expression of NRF2 was downregulated. This is because YTHDF2 binds to m^6^A-methylated *NRF2*, promoting the degradation of its mRNA. The antioxidant effect mediated by NRF2 can alleviate cerebral I/R injury [154].

IGF2BP2 plays an important role in maintaining the function of hematopoietic stem cells (HSCs). The absence of IGF2BP2 accelerates the decay of Bmi1 polycomb ring finger oncogene (*Bmi1*) mRNA, thus increasing mitochondrial activity in HSCs. High mitochondrial activity indicates a lower reconstitution ability of HSCs. Furthermore, the absence of METTL3 increases the expression of mitochondrial-related genes and reduces the expression of genes involved in HSC maintenance. All of these indicate that IGF2BP2 plays a role in inhibiting mitochondrial activity in HSCs [155].

METTL3 can improve mitochondrial dysfunction by increasing the m^6^A modification level of *MFN2* mRNA and enhancing the expression of MFN2. The overexpression of METTL3 or MFN2 can improve mitochondrial dysfunction in Alzheimer’s disease (AD) by, for instance, reducing mitochondrial damage and increasing ATP levels [156]. TRAF6 is a multifunctional signaling molecule belonging to the TRAF family, which performs physiological functions through the Toll-like receptor 4 (TLR4) signaling pathway. The evolutionarily conserved signaling intermediate in Toll pathways (ECSIT) is also a multifunctional protein partially located in the IMM and participates in the assembly of OXPHOS complex I. It has been reported that TRAF6, after rapid activation, translocates to the mitochondria and increases the production of mitochondrial reactive oxygen species (mROS) in macrophages. ECSIT can interact with TRAF6 to participate in the production of mROS and the formation of complex I. The METTL3-mediated m^6^A modification of *TRAF6* activates TRAF6 and causes it to translocate to the mitochondria, activating the TRAF6/ECSIT pathway and leading to the production of mROS. When METTL3 is inhibited, the translocation of TRAF6 to the mitochondria, the expression of ECSIT, and the production of mROS are all weakened, while the levels of ATP and ΔΨ_m_ in the mitochondria increase [157]. Mitochondrial lon peptidase 1 (LONP1) is a protein quality control protease that plays an important role in regulating mitochondrial protein homeostasis and maintains the integrity of mtDNA by selectively degrading abnormal and oxidatively damaged proteins [158]. It has been found that as the kidney ages, the expression of LONP1 and TFAM is significantly reduced, and the levels of mtDNA and ATP are also significantly reduced. Moreover, the mitochondrial structure changes, with mitochondrial swelling and disordered cristae. Studies have found that after overexpressing METTL3, the expression of LONP1 and TFAM significantly increased and the expression of DRP1 reduced, the levels of mtDNA and ATP increased, and the integrity of the mitochondrial structure was maintained; above all, mitochondrial dysfunction was alleviated [159]. A high expression of METTL3 was found in sepsis-induced acute lung injury, which increased the level of m^6^A modification. IGF2BP3 recognizes these modifications and enhances the stability of the mRNA of certain genes, such as *HIF-α*, leading to the abnormal activation of the mitochondrial metabolic pathway, downregulation of GPX4, and exacerbation of ferroptosis, thus aggravating lung tissue injury [160]. Both METTL3 and YTHDF2 are highly expressed in fibrotic cardiac tissues. Growth arrest-specific 5 (*GAS5*) is a long non-coding RNA (lncRNA) located in the mitochondria and regulates the mitochondrial metabolism. GAS5 can bind to DRP1 and inhibit the expression of DRP1. In addition, the expression of GAS5 is regulated by m^6^A methylation. A high expression of METTL3 increases the m^6^A modification of *GAS5*. YTHDF2 induces the degradation of *GAS5* mRNA by recognizing the m^6^A sites on *GAS5*, resulting in increased mitochondrial fission and leading to cardiac fibrosis [161].

The absence of YTHDF1 leads to a decrease in the level of mitochondrial ATP production, a reduction in the expression levels of PGC-1α, TFAM, and MFN2, and an increase in the expression levels of DRP1 and FIS1. This results in a decrease in mitochondrial biogenesis and mitochondria damage, such as fragmentation, swelling, and loss of cristae. Overall, it leads to mitochondrial dysfunction, promotes oxidative stress, and finally causes cell apoptosis [162]. In metabolic-dysfunction-associated steatotic liver disease (MASLD), the expression level of METTL3 increases while the expression level of YTHDF1 decreases. The increased expression of METTL3 promotes the function of the mitochondrial ETC, and the level of energy metabolism also increases significantly. However, the decreased expression of YTHDF1 reverses the effect of METTL3 and inhibits mitochondrial OXPHOS. This is mainly because the m^6^A modification of mitochondrial subunits mediated by METTL3 requires YTHDF1 as a “reader” to stabilize the expression of these RNAs, thus promoting the expression of mitochondrial complexes [163].

Studies have found that the Notch signaling pathway can restore mitochondrial dynamics [164]. ALKBH5 increases the stability of notch receptor 1 (*NOTCH1*) mRNA, leading to the upregulation of NOTCH1 expression and downregulation of DRP1 expression, thereby reducing mitochondrial fission. However, when the m^6^A modification level of *NOTCH1* mRNA increases, it enhances the recognition of m^6^A modification on *NOTCH1* mRNA by YTHDF2, inducing the degradation of *NOTCH1* and promoting mitochondrial fission [165]. ALKBH5 is significantly downregulated in fibrotic liver tissues, while YTHDF1 is significantly upregulated. ALKBH5 demethylates the m^6^A modification on the 3′ UTR of *DRP1*. The downregulation of ALKBH5 leads to the upregulation of m^6^A modification. YTHDF1 promotes the expression of DRP1 by recognizing the m^6^A modification on the 3′ UTR of *DRP1*, thus promoting mitochondrial fission and leading to liver fibrosis [166].

During myocardial ischemia/reperfusion injury (MIRI), various mitochondrial dysfunctions occur, including an impaired mitochondrial ATP production, decreased mitochondrial membrane potential, and excessive production of ROS, resulting in conditions such as myocardial dysfunction, DNA damage, and apoptosis. LncRNA *Snhg1* can upregulate the expression of OPA1 by sponging miR-361-5p, thereby improving the progression of MIRI. However, in MIRI, the expression of lncRNA *Snhg1* is found to be downregulated. This is because WTAP induces the m^6^A modification of lncRNA *Snhg1*, and YTHDF2 recognizes and binds to lncRNA *Snhg1* through its m^6^A modification, thus promoting its degradation [167].

In conclusion, we can observe that although methyltransferases and demethylases play crucial roles in the regulation of diseases, whether m^6^A modification can exert its functions mainly depends on the roles of readers. Different readers mediate different processes, and whether an m^6^A-modified mRNA is upregulated in expression or degraded depends on which reader mediates this process.

### 3.2. Role of m^6^A in Influencing Mitochondrial Function in Cancer

Many studies have found that the growth of tumor cells can be regulated by mitochondrial metabolic reprogramming. Next, we will summarize how m^6^A methylation affects cancer through mitochondria function. Uncontrolled cell proliferation is one of the hallmarks of cancer. To maintain the ability of rapid proliferation, cancer cells make certain adjustments to energy metabolism to obtain more energy—that is, enhancing or activating metabolic pathways [168]. Metabolic reprogramming has become one of the hallmarks of cancer and is also one of the abilities acquired during malignant transformation [169]. Warburg discovered that in the presence of oxygen, cancer cells limit energy metabolism by activating glycolysis and converting glucose into lactic acid, which is known as the Warburg effect [170,171]. Although mitochondrial dysfunction in cancer cells can lead to this shift in energy metabolism, studies have shown that many cancer cells have fully functional mitochondria and are able to oxidize glucose through OXPHOS [172]. The efficiency of ATP production via glycolysis is much lower than that of OXPHOS. To eliminate this efficiency difference, the glucose uptake is increased by upregulating glucose transporters, and glycolysis is enhanced, leading to the accumulation of glycolytic intermediates. These glycolytic intermediates are transferred to different biosynthetic pathways, including pathways for nucleotide synthesis and pathways for generating amino acids and NADPH, to meet the requirements of cell proliferation [173,174]. During the development of cancer, mitochondria promote typical characteristics such as metabolic reprogramming, continuous proliferation, evasion of cell death, invasion, and the induction of angiogenesis (Table 3). Therefore, mitochondria are key mediators of tumorigenesis [175].

Methylenetetrahydrofolate dehydrogenase 2 (MTHFD2), a mitochondrial enzyme, is a key enzyme in the one-carbon metabolic pathway; research has found that the expression of MTHFD2 is significantly upregulated in renal cell carcinoma (RCC). It specifically increases the methylation level of METTL3-dependent hypoxia-inducible factor 2α (*HIF-2α*), promotes the translation of *HIF-2α* mRNA, and increases the protein level of HIF-2α, thus promoting the glycolysis of tumor cells and tumor progression [217].

Von Hippel–Lindau (VHL) deficiency or mutation is frequently observed in clear cell renal cell carcinoma (ccRCC). The loss of VHL function promotes tumorigenesis. Peroxisome proliferator-activated receptor gamma coactivator (PGC), as a family of transcriptional coactivators, mediates mitochondrial biogenesis and OXPHOS. Its family members include PGC-1α, PGC-1β, and PRC. PGC-1α, as a core regulator of mitochondrial function, plays a crucial role. Its expression inhibits tumor growth. FTO has an anti-cancer effect in cancer development and is downregulated in ccRCC. Studies have found that Von Hippel–Lindau-deficient cells expressing FTO reduced the m^6^A level of *PGC-1α* mRNA, stabilizing *PGC-1α* mRNA, restoring mitochondrial activity, promoting oxidative stress and ROS production, and thus inhibiting tumor growth [218].

Gene NADH dehydrogenase (ubiquinone) 1 alpha subcomplex 4 (*NDUFA4*) encodes a subunit in the ETC complex of the mitochondrial respiratory chain and is highly expressed in gastric cancer (GC). NDUFA4 can promote the glycolysis and oxidative metabolism of GC cells, inhibit ROS levels in GC cells, and promote mitochondrial membrane potential (MMP) levels. Moreover, it has been found that METTL3 can increase the m^6^A level of *NDUFA4* mRNA through IGF2BP1 to promote the expression of NDUFA4 in GC cells, thus promoting the development of GC [219].

Studies have found that in cervical cancer and liver cancer cells, when Mettl3 is absent or ALKBH5 is overexpressed, glucose consumption, lactate production, and ATP generation are all inhibited, but there is no significant effect on mitochondrial DNA content. Mechanistically, m^6^A positively regulates glycolysis and ATP generation in cancer cells through pyruvate dehydrogenase kinase 4 (PDK4). The 5′UTR of m^6^A-modified *PDK4* binds to the YTHDF1/eEF-2 complex and IGF2BP3, positively regulating its mRNA stability and translation elongation and thereby affecting the energy metabolism process of cells and indirectly influencing tumor growth and progression [220].

M^6^A can promote the development of colorectal cancer (CRC) by inducing the synthesis of GSH and stabilizing *OPA1* mRNA to promote mitochondrial fusion. Mechanistically, METTL3 modifies ribonucleotide reductase regulatory TP53 inducible subunit M2B (*RRM2B*) and *OPA1* mRNA with m^6^A, while IGF2BP2 increases the stability of mRNA by binding to RRM2B and OPA1, increasing the expression of their proteins and thereby promoting GSH synthesis and mitochondrial fusion [221].

Caveolin 1 (CAV1) is a membrane protein that is essential for maintaining mitochondrial structure and function and is associated with mitochondrial quantity and bioenergetic function. Its role in tumors is controversial. Studies have found that FTO is highly expressed in gastric cancer tissues. The knockdown of FTO inhibits the proliferation, migration, and invasion of gastric cancer cells. Mechanistically, FTO promotes the degradation of *CAV1* mRNA by reducing its m^6^A modification level, thereby affecting mitochondrial fission, fusion, and metabolism, promoting the development of GC [222].

Mitochondrial fission promotes the progression of glioma. Mechanistically, METTL3 promotes the alternative splicing (AS) of *LINC00475* to generate *LINC00475-S* by increasing the binding of m^6^A recognition protein HNRNPH1 to *LINC00475*. By inhibiting the expression of macrophage migration inhibitory factor (MIF), it enhances the expression of DRP1 and p-DRP1 while simultaneously inhibiting the expression of OPA1 and MFN2 to promote mitochondrial fission, thereby promoting the progression of glioma [223].

Research has found that FTO is upregulated in breast cancer and promotes the development of breast cancer. Mechanistically, FTO downregulates BNIP3 through demethylating m^6^A in the 3′ UTR of *BINP3* mRNA and inhibits the apoptosis induced by it, thus promoting the proliferation of breast cancer cells, and this process is independent of YTHDF2 [224].

In papillary thyroid cancer (PTC), the expression of FTO is significantly downregulated. Apolipoprotein E (*APOE*) is an m^6^A modification target downstream of FTO; its m^6^A modification is recognized and mediated by IGF2BP2. The downregulation of FTO promotes the expression of APOE and enhances glycolysis in PTC, thus promoting tumor growth. Mechanistically, FTO acts as a negative regulator of PTC and inhibits the expression of APOE. These changes inhibit glycolysis in PTC by regulating the IL-6/JAK/STAT3 signaling pathway, thereby affecting tumor growth [225].

A high expression of METTL3 is found in chemoresistant small cell lung cancer (SCLC) cell lines and promotes chemoresistance in SCLC. *DCP2* is a downstream target of METTL3; the highly m^6^A-modified *DCP2* leads to the degradation of its mRNA through the selective recognition of YTHDF2. The decreased expression of DCP2 protein enhances the stability of *PINK1* and *Parkin* mRNA, resulting in increased mitophagy and reduced mitochondrial damage. Eventually, it leads to the chemoresistance of SCLC to chemotherapeutic drugs [226].

In CRC cell lines, IGF2BP2 is highly expressed and lncRNA *ZFAS1* is significantly highly expressed. IGF2BP2 promotes the stability of *ZFAS1* through the m^6^A sites on *ZFAS1*, thus promoting the development of CRC. Mechanistically, obg-like ATPase 1 (OLA1) serves as a key downstream target. ZFAS1 binds to the OBG-type domain of OLA1 (Table 4), thereby exposing the ATP binding site on OLA1, enhancing its ATP hydrolysis ability, and activating the glycolytic pathway, thus promoting the development of CRC [227].

In GC tissues, an upregulated expression of METTL3 and IGF2BP3 is found, leading to an increase in the m^6^A modification on *DRP1*. IGF2BP3 recognizes the m^6^A sites and increases the expression of *DRP1* mRNA, thus exacerbating mitochondrial fission, dysfunction, and the increase in mROS, inducing the activation of the NLRP3 inflammasome and promoting the development of gastric cancer [228].

IGF2BP2 is highly expressed in acute myeloid leukemia (AML). It mainly participates in glutamine (Gln) metabolism in AML by enhancing the expression of GPT2 and SLC1A5 in the Gln metabolic pathway. Among them, *GPT2* encodes an enzyme that catalyzes the reversible conversion of pyruvate and glutamate (Glu) into alanine and α-ketoglutarate (αKG) in mitochondria during Gln metabolism, and *SLC1A5* encodes the main transporter of Gln in cancer cells. IGF2BP2 recognizes and increases the mRNA stability and expression of *MYC*, *GPT2*, and *SLC1A5* through m^6^A modification, providing fuel for the TCA cycle, thus promoting the development of AML [229].

**Figure 3:**
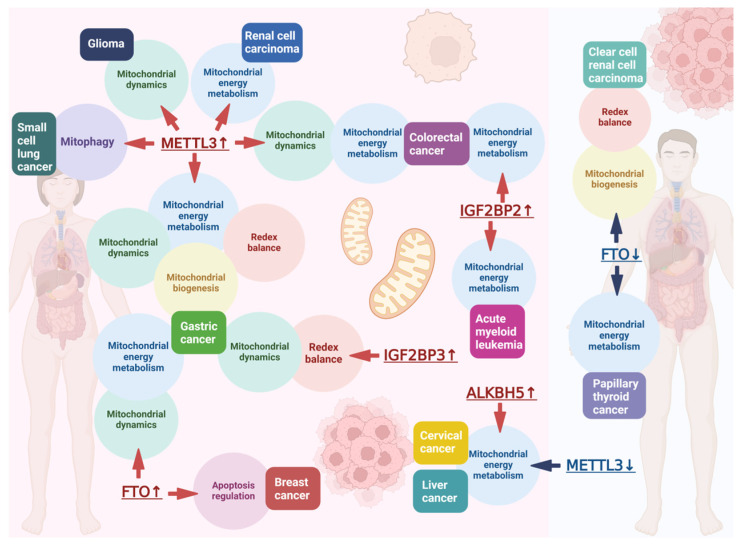
Role of m^6^A in influencing mitochondrial function in cancer (created with BioRender.com). Dysregulated expression of m^6^A regulators (upregulation or downregulation) in tumors modulates cancer progression by affecting mitochondrial functions, including mitochondrial energy metabolism, biogenesis, redox homeostasis, dynamics, mitophagy, and apoptosis regulation. In this figure, the red upward arrow behind the m^6^A regulators indicates upregulation, and the blue downward arrow indicates downregulation.

From the above examples, we can see that the development of cancer is complex. There may be two or even multiple regulatory processes in the same type of cancer. The same demethylase, such as FTO, plays different functions in different cancers. It is a positive regulator in breast cancer but a negative regulator in papillary thyroid cancer. M^6^A methylation plays a dual role in cancer treatment. Both the excessive modification and lack of modification of m^6^A may lead to the development of cancer (Figure 3).

**Table 4:** A summary of the factors that change in diseases.

Factor	Full Name	Function	Reference
TRAF6	Tumor necrosis factor receptor-associated factor 6	Belonging to the TRAFs family, an adaptor protein is recruited to the intracellular region when activated and executes a variety of physiological functions through the TLR4 signaling pathway.	[157]
ECSIT	Evolutionarily conserved signaling intermediate in Toll pathways	A multifunctional protein partially located in the IMM, which participates in the assembly of oxidative phosphorylation complex I.	[157]
LONP1	Lon peptidase 1	A protein quality control protease that plays an important role in regulating mitochondrial protein homeostasis and maintains the integrity of mtDNA by selectively degrading abnormal and oxidatively damaged proteins.	[158]
MTHFD2	Methylenetetrahydrofolate dehydrogenase 2	A mitochondrial enzyme encoded by the nucleus, which participates in folate metabolism and one-carbon metabolism in mitochondria and maintains intracellular redox balance.	[230]
NDUFA4	NADH dehydrogenase (ubiquinone) 1 alpha subcomplex 4	Encodes a subunit in the electron transport chain complex of the mitochondrial respiratory chain to generate ATP.	[219]
PDK4	Pyruvate dehydrogenase kinase 4	Promotes the transition from mitochondrial oxidative phosphorylation to glycolysis and regulates glucose metabolism by phosphorylating pyruvate dehydrogenase.	[231]
RRM2B	Ribonucleotide reductase regulatory TP53 inducible subunit M2B	A key subunit of ribonucleotide reductase (RR) plays important roles in DNA repair, replication, oxidative stress, and mtDNA synthesis.	[232]
RR	Ribonucleotide reductase	Catalyzes ribonucleoside diphosphates to deoxyribonucleoside diphosphates and plays an important role in DNA synthesis and repair.	[232]
Caveolin-1	Caveolin-1	A membrane protein that is essential for maintaining the structure and function of mitochondria and is associated with the number of mitochondria and their bioenergetic functions.	[222]
APOE	Apolipoprotein E	A glycoprotein that functions as a lipid transport protein.	[233]
DCP2	Decapping MRNA 2	A major decapping enzyme during 5′ to 3′ mRNA decay, controlling the expression of PINK1 and Parkin to regulate mitophagy and the level of mitochondrial damage.	[226]
OLA1	Obg-like ATPase 1	An ATP hydrolase that mediates mitochondrial energy metabolism, including ATP hydrolysis and glycolysis.	[227]

### 3.3. Small Molecule Drug Therapy

STM2457 acts as an inhibitor of METTL3. The high expression of METTL3 promotes the degradation of *DCP2* and increases the expression of PINK1 and Parkin, thereby enhancing mitophagy and contributing to chemoresistance in SCLC. STM2457 increases the expression level of DCP2 and decreases the expression levels of PINK1 and Parkin by inhibiting the expression of METTL3 in SCLC cells, thus reversing the chemoresistance of SCLC cells [226].

CWI1-2 serves as an inhibitor of IGF2BP2, binding to the RNA binding site of *IGF2BP2* and competitively inhibiting its binding to other RNAs. Studies have found that CWI1-2 exhibits antileukemic efficacy both in vivo and in vitro. It mainly inhibits the role of IGF2BP2 in AML, reduces the uptake of Gln, impairs mitochondrial function, and decreases ATP production, thus inhibiting the progression of AML [229].

Quercetin (QUE) is a natural flavonoid with properties such as regulating mitochondrial biogenesis and anti-cancer and anti-inflammatory effects. Research has shown that QUE interacts with METTL3. QUE significantly inhibits the expression level of METTL3. In lipopolysaccharide (LPS)-induced liver injury, the use of QUE reduces the m^6^A methylation level of *PTEN*, promotes the activation of the PI3K/AKT signaling pathway, decreases the expression of DRP1, and increases the expression of MFN2, MFN1, and OPA1 as well as key enzymes related to glycolysis and the TCA cycle, effectively preventing the imbalance of mitochondrial dynamics and energy metabolism induced by LPS [234]. In addition, another study found that QUE reduces the m^6^A modification of serine–threonine kinase protein kinase D2 (*PRKD2*) by inhibiting the expression of METTL3 and improves insulin resistance (IR) by promoting glucose uptake and inhibiting oxidative stress [235].

From the above, we can see that small molecule inhibitors targeting m^6^A regulators also hold potential in treating diseases by targeting mitochondrial function. However, when using small molecule inhibitors, attention should be paid to the dosage and whether they are specific. It is also important to develop targeted delivery systems.

## 4. Summary and Prospects

In recent years, it has been found that m^6^A plays an important role in maintaining the normal function of mitochondria, and its abnormality often leads to mitochondrial dysfunction. It plays its role mainly through several aspects: regulating the expression of mitochondria-related genes, influencing mitochondrial biogenesis, regulating mitochondrial dynamics, participating in the oxidative stress reaction, and affecting mitophagy. Readers determine the fate of mitochondria-related genes by recognizing the m^6^A sites on them. For example, some readers will promote the degradation of the mRNA of mitochondria-related genes with m^6^A modification, while other readers will increase the stability of the mRNA and promote translation. M^6^A affects the quality control of mitochondria by participating in the expression of genes related to mitochondrial biogenesis and mitophagy. M^6^A may affect the processes of mitochondrial fission and fusion by regulating the expression of proteins related to mitochondrial dynamics, thereby influencing mitochondrial function. Oxidative stress is a significant cause of mitochondrial dysfunction. M^6^A modification can regulate the expression of genes related to the antioxidant defense system in cells and affect the response ability of cells to oxidative stress. When mitochondria are seriously damaged, and mitophagy fails to remove the damaged mitochondria in a timely manner, it will trigger cell death. M^6^A is involved in the regulation of mitochondrial function at multiple levels, and its abnormality is closely related to mitochondrial dysfunction.

Mitochondrial dysfunction plays a certain role in promoting the development of various diseases. Each disease will create a specific physiological environment. It is crucial to identify specific therapeutic targets according to the physiological characteristics of the disease and different m^6^A-mitochondrial function processes. Secondly, tumor cells meet the requirements of their rapid proliferation through metabolic reprogramming. M^6^A modification can affect the growth and proliferation of tumor cells by regulating the mRNA stability and translation efficiency of key enzymes involved in OXPHOS and glycolysis. M^6^A can also affect mitochondrial fission, fusion, and quality, thereby influencing the progression of cancer by affecting the expression of proteins related to mitochondrial dynamics, biogenesis, and mitophagy. M^6^A plays a complex but important role in cancer by influencing mitochondrial function. Further research on the relationship between m^6^A and mitochondrial function is expected to provide new targets and strategies for cancer treatment.

## Figures and Tables

**Table 3:** Some functional units of mitochondria become abnormal in cancer.

Mitochondrial Function	Abnormalities of Functional Units	Cancer Type	Reference
Mitochondrial energy metabolism	Isocitrate dehydrogenase (IDH) mutant	Gliomas, acute myeloid leukemia, cholangiocarcinoma, chondrosarcoma	[176,177]
	Upregulation of wild-type IDH2	Triple-negative breast cancer, lung cancer, esophageal squamous cell carcinoma	[178,179,180]
	Upregulation of PGC-1α	Triple-negative breast cancer, cholangiocarcinoma	[181,182]
Mitochondrial calcium homeostasis	Upregulation of MCU and downregulation of MICU1	Colorectal cancer, hepatocellular carcinoma, breast cancer	[183,184,185]
	Upregulation of MCUR1	Hepatocellular carcinoma	[186]
	Downregulation of MCU	Prostate cancer, colon cancer	[187]
	Upregulation of VDAC	Breast cancer, head and neck cancer, lung adenocarcinoma	[188]
	Upregulation of VDAC1	Breast cancer	[189]
Mitochondrial dynamics	Upregulation of DRP1	Esophageal squamous cell carcinoma, pancreatic cancer, head and neck cancer, breast cancer, hepatocellular carcinoma	[190,191,192,193,194]
	Upregulation of DRP1 and downregulation of MFN1	Hepatocellular carcinoma, breast cancer	[195,196]
	Upregulation of DRP1 and downregulation of MFN2	Lung cancer	[197]
	The mitochondrial protein FUNDC2 inhibits MFN1	Hepatocellular carcinoma	[198]
	MFN1 frameshift mutations	Colorectal cancer	[199]
	Downregulation of MFN2	Breast cancer, lung cancer, bladder cancer	[200,201]
	Upregulation of OPA1 and MFN1	Lung adenocarcinoma	[202]
	Upregulation of fission factor	Hepatocellular carcinoma	[203]
	Upregulation of FIS1	Oral melanoma, acute myeloid leukemia	[204,205]
Mitophagy	High expression of mucin 1 (MUC1) protects PINK1 from cleavage, thereby increasing mitochondrial autophagy	Breast cancer	[206]
	Upregulation of sequestosome 1 (SQSTM1/p62)	Hepatocellular carcinoma	[207]
	Upregulation of PINK1	Esophageal squamous cell carcinoma, non-small cell lung cancer	[208,209]
	Downregulation of Pankin	Colorectal cancer, clear-cell renal cell carcinoma, oropharyngeal squamous cell carcinoma, pancreatic ductal adenocarcinoma	[210,211,212,213]
	Upregulation of BNIP3	Renal cell carcinoma	[214]
	Upregulation of FUNDC1	Breast cancer, cervical cancer	[215,216]
